# On the State of the Nutritive Functions during the Progress of Continued Fever

**Published:** 1859-11

**Authors:** Bedford Brown

**Affiliations:** Yanceyville, Caswell co., N. C.


*On the state of the Nutritive Functions during the progress of Continued Fever.* By Bedford Brown, M. D., of Yanceyville, Caswell co., N. C.

According to present opinion, the absorption into the system of a certain morbid poison is the prime cause of continued fever. Admitting this to be true, it is still questionable whether it is the sole and immediate cause of all of the pathological changes and phenomena which arise during the progress of that disease. On the absorption of this poison into the circulation, a deleterious impression is first made, we believe, on the blood. The essential nature of that impression is an impairment of the vital and nutritive properties of that fluid. This may be considered the primary stage of typhoid fever.

There are also changes of a secondary character, which are not only the immediate cause of some of the most important pathological phenomena of the disease, but in reality exert a controlling influence over the course and termination of it. The true nature of these changes is imperfect nutrition of the organised tissues.

The mental depression, the physical prostration, the remarkable decline of the organic functions, and the rapid emaciation, all afford abundant proof of the serious impairment which the nutritive and assimilative powers have sustained from the very beginning of the attack. Many of the leading symptoms or indications of morbid conditions, either of a functional or organic character, occurring in fever, are not peculiar to it. These identical symptoms are equally as common in the course of many other diseases. The integrity of the intellectual and physical powers is known to depend on certain regular and constant molecular changes which the tissues undergo in the assimilative process. These changes consist in a well-balanced process of renewal and destruction of structure. Now, when this balance is disturbed, in either the general assimilation or the nutrition of any particular organ, functional and organic derangement must be the consequence. Every action of an organ, whether of an organic or animal nature, causes that organ to sustain a loss of a certain amount of material by disintegration, and without a compensating renewal of matter, it would be no longer capable of acting again, or would act imperfectly. Now, many of the morbid phenomena of continued fever are in truth due, as an immediate cause, to the defective renewal of material to sustain the molecular condition of the different tissues. These phenomena consist of organic changes within the tissues, derangement or impairment of the various functional operations of the individual organs, and of the entire organism. To produce these effects, there is a double cause in operation in typhoid fever. First, on the absorption of the morbid poison, which generates the disease, the nutritive properties of the blood are primarily affected.—They may be simply impaired to a certain extent, or so far destroyed as to suspend the nutritive process instantaneously and cause immediate death. Again, the digestive organs, being imperfectly nourished, fail to elaborate proper materials for assimilation. It is a remarkable fact, that in all of those blood diseases, in which the nutritive operation is greatly impaired, the structure of the various organs and tissues are found in a softened condition after death. This cannot be accounted for, otherwise, than by the supposition that proper material has not been regularly supplied for interstitial renovation.

To substantiate these opinions, it will be necessary to go into an investigation of the physical condition of the blood in fever, and of the influence exerted on the interstitial condition of the individual parts of the organism, by a deficient supply of nutritive matter for assimilation, together with the special symptoms arising from such a state in the form of deranged or suspended functions.

I believe that a correct understanding of this subject has a most important bearing on the management and treatment of the disease; so much so, that it is well worthy of the labor of a thorough investigation. Indeed, with our old notions of "Nervous Irritation," "Excitement," &c., and the false importance attached to them as chief elements in the pathology of fever, I consider, while entertaining such views, that we are totally unqualified to manage successfully a severe case of this disease.

*Physical Condition of the Blood in Typhoid Fever.*—According to analysis by the best authorities, the nutritive properties of the blood are diminished in continual fever, particularly the amount of fibrin. The diminution dates from the commencement, and increases not only as the disease advances, but in proportion to the severity of the attack. In the latter stages of severe cases, the blood becomes thin, poor, and greatly deficient in solid material. It is probably the fibrinous or plastic materials, of all others, are the most important for the support and nutrition of the human fabric. This diminution is not limited to quantity. The vital qualties are equally impaired. This is plainly manifest by the difficulty with which the blood sustains the vital action of resolving inflammations, and of healing wounds and ulcers. There is also a corresponding decrease in the quantity of salts in the blood of typhoid patients.

*Effects of Innutrition on the Condition of the Nervous System in Fever.*—In severe cases of continued fever, delirium, subsultus, vigilance, finally somnolence and coma, are always present. These symptoms are not peculiar to typhoid, nor even to fevers of a kindred nature, but are common to all diseases in which there is a deficiency in supply of properly elaborated materials, for the constant renovation of the nervous mass. In the simpliest of all forms of innutrition, that of starvation, wherein all food is withheld from the system, these identical symptoms are developed to a prominent degree.

The brain has failed to receive its usual and proper quantity of albumen, phorus, fatty matter, &c., for the renewal of its structure, and consequently the functions proper to it become unsteady and irregular, and finally end in total cessation, when nutrition has been suspended.

The physiological principle that every action of an organ causes it to sustain a loss of a certain amount of substance by disintegration, and without a corresponding or compensating renewal of matter, the same organ would no longer be capable of acting again, is admirably illustrated in this case.

In the early stages of fever, when the nutritive process is but slightly impaired, the functions of the brain are not as vigorous as in health, the mind is vacillating, and the will is unsteady and not as capable of directing as usual. There is an unusual gloom and depression hovering over it, or there is an indifference to consequences and a disposition to surrender. Nor will the muscular powers respond to the mandates of the will, as common. Here is plainly a want of nervous vigor and strength, induced by a slight impairment of interstitial nutrition. I have no doubt that the nutritive operation begins to decline as soon as the morbid poision of fever is absorbed. In proportion as this operation is interfered with, the course of the disease will be rapid and violent. About the second or third week of fever, when the nourishing properties of the blood are becoming seriously damaged, and the integral renewal of nervous matter is particularly suspended; then it is, that what was early in the attack but slight falterings of the nervous energies, become marked failures of organic action, which gradually merge into delirium, subsultus, then somnolence, and finally coma, telling the truth too plainly that the functions of the brain are abolished, because there is no longer material in the blood for the renovation of tissue, and the supply of waste which the organ has sustained.

The peculiar nervous symptoms of delirium tremens I consider a good example of the influence exerted on the functions of the brain, from impaired nutrition and waste of nervous matter. That the nutritive properties of the blood and nutrition of the tissues suffer greatly from constant imbibition of alcohol is not doubted.

After exhausting hemorrhages from any cause, the supervention of nervous symptoms is an invariable consequence. Here we witness violent delirium, convulsions, muscæ volitantes, and coma, originating from a cause that cannot be mistaken, which consists absolutely in a non-supply of nutritive material for the sustenance of the nervous system. Whilst the defect in this case is due to quantity of material—in the case of continued fever the quality is equally at fault. The effects, organically considered, are very much the same. The human system can be starved to death as readily by altering the quality of the blood, as by reducing the quantity.

What is termed "False Hydrocephalous," is another instance of extreme derangement of the nervous system from want of sustenance. In this peculiar disease, all of the nervous symptons are present that usually attend acute inflammation of the brain. Yet after death not a trace of organic lesion can be discovered; on the contrary, the brain is found pale, wasted, and softer than natural. During the protracted progress of chronic indigestion, a great variety of nervous symptoms arise, though differing somewhat from the foregoing. The moral and mental vacillation of the dyspeptic: his variable temper—his whims and caprices—the extreme excitability of his nervous system—his tendency to melancholy, and finally to confirmed lunacy, are all substantial evidence that his brain is suffering from the want of a properly elaborated supply of nutritive matter. A large portion of the ingesta taken into the stomach in dyspepsia, when not rejected by that organ, or passed through the intestines in an unaltered form, is so improperly reduced and converted, that it is rendered unfit for purposes of assimilation. Hence this material, instead of being appropriated for the renovation of tissue, after floating about in the circulation, is eliminated as waste matter, much of it from the kidneys in the form of lithates, phosphates, &c.

I have deemed it necessary to produce these cases as evidences, not simply confirmatory of the fact that symptoms of nervous derangement are a common sequence of imperfect nutrition, but to establish the equally important fact that such symptoms are not confined to continued fever, but are equally as frequent in other diseases in which defective nutrition is a prominent element. Again, I wish to combat the idea that nervous deragement is attributable, in fever, merely to "irritation" or "sympathy," both vague and indefinite terms. Neither can the nervous derangement of fever be the immediate result simply of the impression of the specific cause of that disease, for such derangement rarely occurs until that stage when the general nutrition is beginning to fail. At the same time such symptoms are equally as common in diseases which the morbid poison of fever is entirely absent. The correct comprehension of this subject has a most important practical bearing.

*Condition of the Circulatory Organs in Typhoid Fever.*—The heart, of any single organ, has one of the most difficult parts to sustain, not only in fever, but in all other diseases. On it devolves the duty of sending the vital fluid to the remotest parts of the body, and thence receiving it in retnrn, discharging it into the pulmonic system, to be subjected to atmospheric action. The great fatality of continued fever is not at all astonishing when we consider, in connection with these important and necessary duties, what a vast influence any impairment or withdrawal of the adequate amount of nutritive material has on the heart's action. A decline in the heart's action in fever assumes an importance paramount to all other considerations. If the principle that every time an organ is brought into action it sustains a certain loss of material, by oxidation, holds good in other instances, it is especially so in regard to the heart in typhoid fever. That organ, so long as life remains, must continue in operation unceasingly, in this particular differing from all other organs. When we know that, in common with other parts, in fever it fails to receive the due proportion of nutritive material for the renovation of its tissues, and, at the same time, that this tissue is constantly undergoing a disintegrating process and waste caused by rapid and incessant action, the powers of the organ to maintain the all-important duties devolving on it, are truly wonderful. Doubtless the failure of the heart's action in asthenic cases, is due to the fact that there is not a sufficient renewal of material to supply the waste of cardiac tissue and sustain its vital action.

In some of the most malignant cases of fever that I have ever witnessed, the heart's action was extremely feeble and slow. No amount of artificial stimuli could arouse it to increased action. These cases terminated more rapidly in death than those in which the circulation was greatly increased. Indeed, the explanation of this seems to me to be the utter failure of the nutrition of the substance of the heart. The disintegration and waste of the cardiac substance caused by a pulsation of a hundred and forty or more to the minute, must draw heavily on the heart, and, without a compensating renewal of the material, such increased action must of necessity result in softening of the tissue of the organ, and exhaustion of its powers. In my opinion, herein consists one of the chief dangers to be apprehended from a rapid circulation in fever. Hence the importance of controlling the circulation. Softening of the cardiac tissue is frequently found after death from continued fever, and is probably more frequent than is even supposed. This is a mere fragment of a great constitutional change that is ever in progress in fever, and a certain consequence of imperfect assimilation from impure blood. A loss of affinity between the fluid and solids, in fever, is the probable cause of most of the intercurrent inflammations occurring during the progress of the disease. The healthy circulation through the capillary system is no doubt materially influenced and even regulated, by the condition of the nutritive process. When the integrity of that process has been impaired, a disturbance of the capillary circulation will necessarily follow.—Hence the occasional occurrence of congestions and subacute inflammations in fever.

*Influence that Hemorrhage exerts on the progress of Fever.*—All practical physicians are aware of the danger of hemorrhage as a complication of continued fever, and hail it with dread and apprehension. I believe it can be truly said there are but few diseases in which the body suffers more from the loss of blood than in this. So strictly is this the fact, that but few physicians will resort to bleeding in inflammatory complications. Now, when the nutritive properties of the blood are so materially diminished, as they certainly are in fever, it is not surprising that the occurrence of hemorrhage should exert such a baneful influence over the progress of the attack. There is another difficulty, also. In fever the capacity of the digestive and assimilative organs for the elaboration and formation of new blood, is imperfect. I have, in more than one instance, witnessed what previously was a very mild attack of fever, converted into a most malignant one by a comparatively moderate amount of hemorrhage. To bring forward additional testimony for the purpose of sustaining my opinion, that, namely, many of the phenomena and symptoms of fever are due, as an immediate cause, to a defective amount of nutritive material in the circulation, and an imperfect state of the nutritive process, to cite the agency of hemorrhage in modifying an attack of fever, is almost sufficient of itself. One instance we will select as an example. An attack of fever that has observed a comparatively moderate character up to the supervention of a considerable hemorrhage. Suddenly the entire aspect changes; new features appear, that were absent before; a total perversion of the animal and physical powers takes place; there is delirium, subsultus, and coma, a very rapid and feeble action of the heart, a sudden decline of animal heat, and, in fact, all the signs of the exhaustion of the fountains of nutrition.

*The Accumulation and Escape of Effete Matter in Fevers.*—The degree of diarrhœa is a very good criterion of the amount of effete matter accumulating in the circulation. When the quantity of nutritive matter in the blood is reduced so far below the standard of health, the disintegration of tissue and accumulation of effete matter must be very great. This effete matter doubtless escapes from the system principally through the intestines and skin, in fever. That the diarrhœa of fever is chiefly due to ulceration of intestinal glands, I cannot believe, any more than diarrhœa is the principal element in cholera. There is something more than the elimination of the fluid portions of the blood. There is a great constitutional revolution at work in both instances, in which the molecular structure is rapidly being broke down, and the cell formation and growth is arrested. How can the excessive emaciation and waste of the textures be accounted for, except by the fact that some agent having been introduced, has impaired, or even arrested, the nutrition of the body, by acting on the vitality of the blood? The balance between the formation and the disintegration of structure, is lost in favor of the latter process. The *debris* of broken tissue must find an outlet through some emunctory. No, the causes of typhoid and choleraic diarrhœa, are further back in the depths of the system than any change confined to the intestinal tube could produce. The accumulation of that substance known as "typhoid deposit," in the intestinal glands, is, I believe, nothing more than a portion of the general mass of effete matter in the system, and which is finding an outlet in this manner. But then the copious diarrhœa of fever is not to be encouraged from a false belief that all the effete matter in the circulation is useless, and should be expelled as soon as possible. To the contrary, it is a well-established principle of physiology, that much of this matter is not only not useless, but when retained, is constantly being reassimilated into the solid structure.

*Sloughing and Ulceration during Continued Fever.*—When the healthy nutrition of either a portion or of the entire body has been materially interrupted, the disintegrative absorption of the solids becomes so rapid as to assume the form, in particular localities, of sloughing or ulceration. This state of things is particularly liable to occur in parts in which the circulation is rendered more feeble than natural, either from mechanical or physiological causes. I regard what is termed the bed-sore of fever as a true type of the vital condition of the system, and an accurate representation in miniature of the state of the great nutritive operations going on in the constitution. By it we are enabled to observe on a small scale, these operations in progress, and to learn the important fact that the solid tissues are breaking down and wasting away for the want of a healthy renewal, and that the process of disintegration is in the ascendency. The absorption, in protracted cases of fever, of the osseous unions of old fractures, and of the cicatrices and wounds of ulcers long healed up, afford another instance of the imperfect state of the nutritive functions and the rapid destruction of tissue.—*American Jour. Med. Sciences.*

